# Frequency of the moyamoya-related *RNF213* p.Arg4810Lys variant in 1,516 Korean individuals

**DOI:** 10.1186/s12881-015-0252-4

**Published:** 2015-11-20

**Authors:** Mi-Ae Jang, Sue Shin, Jong Hyun Yoon, Chang-Seok Ki

**Affiliations:** Department of Laboratory Medicine, Korea University College of Medicine, Seoul, 08308 South Korea; Department of Laboratory Medicine, Boramae Hospital, Seoul National University College of Medicine, Seoul, 07061 South Korea; Department of Laboratory Medicine and Genetics, Samsung Medical Center, Sungkyunkwan University School of Medicine, Seoul, 34141 South Korea

**Keywords:** Korean, Moyamoya disease, Odds ratio, p.Arg4810K, *RNF213*

## Abstract

**Background:**

Moyamoya disease (MMD) is a progressive steno-occlusive vasculopathy that involves large intracranial arteries accompanied by abnormal collateral vessels. Recently, *RNF213* was identified as a susceptibility gene for MMD and p.Arg4810Lys (rs112735431) is the most common variant in East Asian MMD patients. Interestingly, many studies have reported that a certain proportion of the general population in Japan, Korea, and China also has this variant. In this study, we investigated the frequency of this variant and estimated an odds ratio of MMD using two different Korean populations.

**Methods:**

A total of 1,516 anonymous DNA samples, 799 from an umbilical cord blood bank and 717 from routine health-checked adults, were genotyped using targeted Sanger sequencing.

**Results:**

The p.Arg4810Lys variant was detected at genotype frequencies of 2.25 % (18/799; 95 % confidence interval (CI), 1.43-3.53 %) in cord blood samples and 2.65 % (19/717; 95 % CI, 1.70-4.10 %) in adult samples, respectively. This variant showed a strong association with MMD (*P* < 0.001), giving an odds ratio of 162.7 (95 % CI, 65.5–403.9) and 137.8 (95 % CI, 55.8–339.9) based on the cord blood and adults samples, respectively.

**Conclusions:**

These results confirm that the *RNF213* p.Arg4810Lys variant is not uncommon in the general Korean population and provide reference data for the association of this variant and MMD.

## Background

Moyamoya disease (MMD; OMIM 607151) is a rare progressive steno-occlusive vasculopathy that involves intracranial arteries accompanied by abnormal collateral vessels [1]. The annual incidence of MMD is estimated to be 0.35–0.94 per 100,000 person-years in Japan [1, 2], and about one-tenth of that in Europe [3]. In Korea, the annual incidence steadily increased from 1.7 to 2.3 per 100,000 person-years from 2007 to 2011 [4].

Recently, Kamada *et al*. identified that *RNF213* as a susceptibility gene for MMD, and p.Arg4810Lys (rs112735431) as a founder variant common in East Asian MMD patients [5]. In affected Asian families, Liu *et al*. showed that the *RNF213* p.Arg4810Lys variant segregated with the disease and demonstrated a predominantly autosomal dominant inheritance pattern with reduced penetrance [6].

Interestingly, the *RNF213* p.Arg4810Lys variant has been found in general populations and the frequency has been estimated to be 0.9–2.7 % [6–8]. Considering the frequency (23.1–90.1 %) of the p.Arg4810Lys variant in MMD, a strong association of this variant and MMD has been suggested, giving an odds ratio of 112–236 [6–8]. In the Korean population, the frequency of the p.Arg4810Lys has been estimated to be 2.69–2.72 % [6, 8]. However, because of the limited numbers of control subjects used in previous studies, further validation of the frequency in a large Korean population is necessary. Therefore, we investigated the frequency of the *RNF213* p.Arg4810Lys variant in two Korean populations.

## Methods

### Subjects

A total of 1,516 anonymous DNA samples were included in this study. Among them, 799 DNAs were obtained from a public umbilical cord blood bank (Seoul Metropolitan Public Cord Blood Bank, Korea) and the remaining 717 DNAs from adult health-examinees were acquired from a hospital-based biobank. All samples were collected from unrelated Koreans who provided informed consent, and the study protocol was approved by the Institutional Review Boards of Seoul National University Boramae Hospital and Samsung Medical Center.

### Genotyping of *RNF213* p.Arg4810K Variant

Exon 60 of *RNF213* was amplified using a primer set designed by the authors: *RNF213*-e60-F, 5’-gctgcatcacaggaaatgac-3’ and *RNF213*-e60-R, 5’-aaggagtgagccgagtttga-3’. The PCR products were sequenced on an ABI 3730*xl* analyzer (Applied Biosystems, Foster City, CA, USA) using a BigDye Terminator v3.1 Cycle sequencing kit (Applied Biosystems). Obtained sequence was compared with the reference sequence for *RNF213* (NM_001256071.1).

### Statistical Analysis

To assess the data quality of *RNF213* p.Arg4810Lys variant, *P*-values were calculated by an exact test for Hardy-Weinberg equilibrium [9]. To analyze genotype and allele frequency data, differences between control and disease groups were compared by a *χ*^2^ or Fisher’s exact test as appropriate. The calculations were performed with VassarStats (http://vassarstats.net/), and 95 % confidence intervals (CI) were calculated for each value. All *P*-values were based on two-sided comparisons, and *P*-values < 0.05 were considered to indicate statistical significance.

## Results

Genotype distributions of *RNF213* p.Arg4810Lys variant in two different sets were in Hardy-Weinberg equilibrium (*P* = 1.00). Of the 1,516 samples tested by sequence analysis, 2.44 % (37/1,516; 95 % CI, 1.78–3.35 %) were heterozygous for the p.Arg4810Lys variant (Table 1). In the subgroup analysis, the p.Arg4810Lys variant was detected at genotype frequencies of 2.25 % (18/799; 95 % CI, 1.43–3.53 %) in cord blood samples and 2.65 % (19/717; 95 % CI, 1.70–4.10 %) in adult samples, respectively. The genotype frequency of the p.Arg4810Lys variant allele was similar between groups. No homozygote was detected in our study. Estimated frequencies of the variant allele were 1.13 % (95 % CI, 0.72–1.78 %) in cord blood samples and 1.32 % (95 % CI, 0.85–2.05 %) in adult samples. In comparison with the data on Korean MMD patients from a published study (30/38; minor allele frequency, 39.5 %) [6], the carrier containing the *RNF213* p.Arg1480Lys variant showed a strong association with MMD in the Korean population (*P* < 0.001), giving an odds ratio of 162.7 (95 % CI, 65.5–403.9) and 137.8 (95 % CI, 55.8–339.9) based on the cord blood and adults samples, respectively.

**Table 1:** Population frequencies of the *RNF213* p.Arg4810Lys variant in Asian countries

Population	c.14429G > A genotype			Sample	Carrier frequency %	MAF %	*P*-value^a^	Reference
(sample type)	Wild-type: G/G	Hetero-zygous: G/A	Homo-zygous: A/A	size	(95 % CI)	(95 % CI)		
Korean (adults)	**698**	**19**	**0**	**717**	**2.65 (1.70–4.10)**	**1.32 (0.85–2.05)**	**-**	**This study**
Korean (cord blood)	**781**	**18**	**0**	**799**	**2.25 (1.43–3.53)**	**1.13 (0.72–1.78)**	**0.76**	**This study**
Korean (unknown)	286	8	0	294	2.72 (1.38–5.28)	1.36 (0.69–2.66)	1.00	[8]
Korean (unknown)	217	6	0	223	2.69 (1.24–5.74)	1.35 (0.62–2.91)	1.00	[6]
Japanese (unknown)	1,437	34	3	1,474	2.51 (1.83–3.44)	1.36 (1.00–1.85)	0.92	[8]
Japanese (unknown)	374	9	1	384	2.60 (1.42–4.72)	1.43 (0.80–2.54)	0.84	[6]
Chinese (unknown)	582	5	0	587	0.85 (0.36–1.98)	0.43 (0.18–1.00)	0.02	[8]
Chinese (unknown)	148	2	0	150	1.33 (0.36–4.73)	0.67 (0.18–2.40)	0.56	[6]

Abbreviations: *MAF*, minor allele frequency; *CI*, confidence interval

The results from this study are shown in bold

^a^Korean (adults) vs other study

## Discussion

Early estimates of the genotype and allele frequencies for *RNF213* p.Arg4810Lys have been reported to be 2.69–2.72 % and 1.35–1.36 % in the Korean population [6, 8], but so far no large-scale population studies have been performed. In this study, we confirmed that the genotype and allele frequencies of the variant were 2.25–2.65 % and 1.13–1.32 %, respectively, which are similar to frequencies in previous reports. The observed frequencies of *RNF213* p.Arg1480Lys variant have also been reported to be similar in proportion to the frequencies reported for the general Japanese population, but the frequencies in the Chinese population are one-thirds less (Table 1).

In the latest report investigating the prevalence of MMD in a Korean population cohort, the total number of patients was 16.1 per 100,000 person-years in 2011 [4], which is a continually growing trend. There are several explanations for the high prevalence of MMD. First, the survey is based on the data from National Health Insurance, which is a nationwide universal insurance system, thus all inpatients and outpatients who were diagnosed with MMD were included. Second, widespread use of non-invasive brain magnetic resonance imaging might increase the detection of asymptomatic MMD in adults.

According to data from the Korean Statistical Information Service (http://kosis.kr/; accessed on 17 December 2014), the total population was 51.42 million in 2014. Based on the frequency of carriers in this study, the total number of carriers was estimated to be 1.25 million. Assuming that MMD occurs in one of the 300 carriers with the *RNF213* p.Arg1480Lys variant [6, 8, 10], the estimated numbers of Korean patients with MMD attributable to *RNF213* p.Arg1480Lys is 4,167. On the basis of the observed prevalence of 16.1 per 100,000 persons, these numbers are the minimum estimates, suggesting that this *RNF213* p.Arg4810Lys variant is not the single risk factor for MMD. Other modifying factors, such as other *RNF213* rare variants, another genetic factor, or environmental factors, might explain the large gap between the prevalence of carrier of the variant allele and the observed prevalence of MMD.

## Conclusions

Our data indicate that the *RNF213* p.Arg4810Lys variant for MMD is not uncommon in the general Korean population, showing a frequency similar to that of the Japanese population. To our knowledge, this study represents the largest Korean cohort to be analyzed for *RNF213* p.Arg4810Lys, and this report will provide reference data for the association of this variant and MMD.

## Abbreviations

MMD: Moyamoya disease
CI: confidence interval
